# Deficient of a Clock Gene, Brain and Muscle Arnt-Like Protein-1 (BMAL1), Induces Dyslipidemia and Ectopic Fat Formation

**DOI:** 10.1371/journal.pone.0025231

**Published:** 2011-09-22

**Authors:** Shigeki Shimba, Tomohiro Ogawa, Shunsuke Hitosugi, Yuya Ichihashi, Yuki Nakadaira, Munehiro Kobayashi, Masakatsu Tezuka, Yasuhiro Kosuge, Kumiko Ishige, Yoshihisa Ito, Kazuo Komiyama, Yuko Okamatsu-Ogura, Kazuhiro Kimura, Masayuki Saito

**Affiliations:** 1 Department of Health Science, School of Pharmacy, Nihon University, Funabashi, Chiba, Japan; 2 Department of Pharmacology, School of Pharmacy, Nihon University, Funabashi, Chiba, Japan; 3 Department of Pathology, School of Dentistry, Nihon University, Tokyo, Japan; 4 Department of Biomedical Sciences, Graduate School of Veterinary Medicine, Hokkaido University, Kita-ku, Sapporo, Japan; 5 Department of Nutrition, School of Nursing and Nutrition, Tenshi College, Sapporo, Japan; University of Padova, Italy

## Abstract

A link between circadian rhythm and metabolism has long been discussed. Circadian rhythm is controlled by positive and negative transcriptional and translational feedback loops composed of several clock genes. Among clock genes, the brain and muscle Arnt-like protein-1 (BMAL1) and circadian locomotor output cycles kaput (CLOCK) play important roles in the regulation of the positive rhythmic transcription. In addition to control of circadian rhythm, we have previously shown that BMAL1 regulates adipogenesis. In metabolic syndrome patients, the function of BMAL1 is dysregulated in visceral adipose tissue. In addition, analysis of SNPs has revealed that BMAL1 is associated with susceptibility to hypertension and type II diabetes. Furthermore, the significant roles of BMAL1 in pancreatic β cells proliferation and maturation were recently reported. These results suggest that BMAL1 regulates energy homeostasis. Therefore, in this study, we examined whether loss of BMAL1 function is capable of inducing metabolic syndrome. Deficient of the *Bmal1* gene in mice resulted in elevation of the respiratory quotient value, indicating that BMAL1 is involved in the utilization of fat as an energy source. Indeed, lack of Bmal1 reduced the capacity of fat storage in adipose tissue, resulting in an increase in the levels of circulating fatty acids, including triglycerides, free fatty acids, and cholesterol. Elevation of the circulating fatty acids level induced the formation of ectopic fat in the liver and skeletal muscle in *Bmal1* -/- mice. Interestingly, ectopic fat formation was not observed in tissue-specific (liver or skeletal muscle) *Bmal1* -/- mice even under high fat diet feeding condition. Therefore, we were led to conclude that BMAL1 is a crucial factor in the regulation of energy homeostasis, and disorders of the functions of BMAL1 lead to the development of metabolic syndrome.

## Introduction

In recent years, the metabolic syndrome has become increasingly prevalent with major public consequences. Various lifestyle changes compared with a few decades ago are suspected to be the cause of the rapid increase in the number of metabolic syndrome patients. These changes include excessive energy intake from lipid-based foods, late dinners, reduced sleeping time, lack of exercise, excessive stress, etc. In addition to these factors, there is accumulating epidemiological evidence that shift work increases the risk of metabolic syndrome [1]–[4]. Tenkanen et al. reported that, in Finland, when all shift workers were compared with all day workers, the relative risk for ischemic heart disease was 1.4-fold greater in the former after adjustment for lifestyle factors, blood pressure, and serum lipid levels [1]. Similarly, the Nurses' Health Study in the United States reported that the multivariate-adjusted relative risks for ischemic heart disease were 1.51-fold higher among women reporting 6 or more years of rotating night shifts, compared with women who had never done shift work [2]. Karlsson et al. reported that high triglycerides (TG) and low concentrations of high-density lipoprotein (HDL) cholesterol seem to cluster together more often in shift workers than in day workers [3]. A large-scale prospective cohort study in Japanese workers showed that, compared with day workers, rotating-shift workers had a significantly higher risk of death due to ischemic heart disease, whereas fixed-night work was not associated with ischemic heart disease or obesity [4].

The mechanism by which shift work increases metabolic syndrome onset risk is not clear, but it is speculated that shift work has an influence on the circadian rhythms of physiologic functions such as blood pressure, heart rate, and the secretion and excretion of hormones, etc. [5]–[11]. The machinery of circadian rhythm has been conserved throughout evolution. In the regulation of circadian rhythms, two transcription factors, the brain and muscle Arnt-like protein-1 (BMAL1; also referred to as MOP3 or Arnt3) and circadian locomotor output cycles kaput (CLOCK), play central roles [12]–[15]. BMAL1 and CLOCK form a heterodimer and drive transcription from E-box elements found in the promoters of circadian-responsive genes, including *period* (*Per*) 1 and *cryptochrome* (*Cry*). After translation of the Per and Cry proteins, the Per/Cry complex translocates to the nucleus, where it inhibits gene expression driven by BMAL1 and CLOCK [16]–[19].

In addition to its roles in the control of circadian rhythms, BMAL1 has been suggested to contribute to the regulation of metabolism for the following reasons. First, genome-wide profiling of BMAL1 targets revealed their strict relationship with metabolism [20]. Second, SNP analysis revealed that BMAL1 is associated with type II diabetes and hypertension [21]. In addition, BMAL1 is strongly induced during adipogenesis and regulates adipocyte functions [22]. BMAL1 activity is disturbed in the visceral adipose tissue of metabolic syndrome patients [23], [24]. A recent study revealed that BMAL1 and CLOCK play critical roles in proliferation and functions of pancreatic β cells [25]. In addition, Hemmeryckx et al. reported that lack of *Bmal1* increases the arterial and venous thrombosis [26]. In regard to food intake, *Bmal1*-deficient (-/-) mice show robust food anticipatory activity [27]. Moreover, mice carrying mutation in the *Clock* gene are obese and develop a metabolic syndrome consisting of hyperleptinemia, hyperlipidemia, and hyperglycemia [28]. These results suggest that loss of functions of BMAL1 may lead to the development of metabolic syndrome.

We show here that global (whole body) *Bmal1* -/- mice, derived from the embryonic stem (ES) cells of C57BL/6J mice, exhibited a metabolic syndrome-like onset, i.e., elevation of the level of circulating fatty acids, including TG, free fatty acids, and low-density lipoprotein (LDL)-cholesterol. In addition, ectopic fat formation was observed in the liver and skeletal muscle. The mechanism of these effects may involve loss of the functions of adipose tissue, since ectopic fat formation was not observed in tissue-specific (liver or skeletal muscle) *Bmal1* -/- mice even under high fat diet feeding condition. Consequently, we are led to conclude that BMAL1 plays crucial roles in the control of energy homeostasis.

## Results

### Loss of the *Bmal1* gene results in an increase of the respiratory quotient (RQ)

In this study, we first generated conditional *Bmal1* (flox/flox) mice carrying the conditional *Bmal1* allele containing exons 6 to 8 flanked by loxP sites (Fig. S1 and S2). Since genetic background-specific phenotypes were observed in *Clock*-mutant mice [28], [29], conditional *Bmal1* (flox/flox) mice were obtained using ES cells derived from C57BL/6J mice, and the genetic background of the mice was maintained on C57BL/6J. These mice are useful to generate both global- and tissue-specific mice. In all experiments, littermate mice were used as a “control”. Similar to the *Mop3* -/- mice established previously [14], the *Bmal1 -/-* mice exhibited loss of circadian rhythm in locomotor activity (Fig. S3). In contrast, locomotor activity of liver-specific (L-) *Bmal1* -/- mice and skeletal muscle-specific *Bmal1* -/- mice showed no significant differences with that of control mice (Fig. S3).

**Figure 1:**
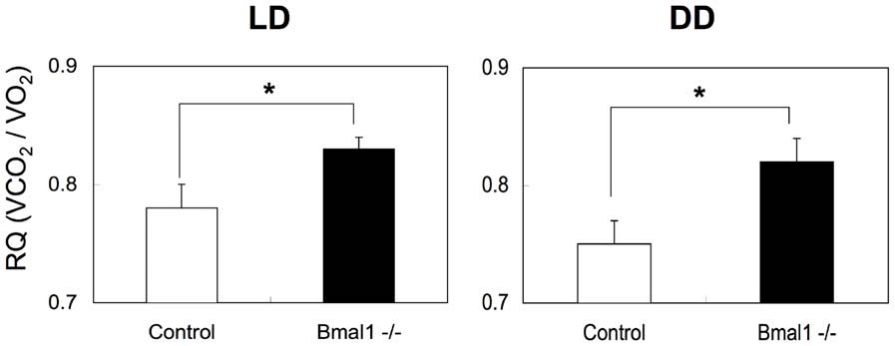
Abnormal respiration activity in *Bmal1* -/- mice. (A) The respiratory quotient (RQ) of male mice housed under the light/dark condition (LD) or constant darkness condition (DD) was determined by indirect calorimeter. The daily average of RQ corrected by body weight was calculated. Data represent the means ± SEM (n = 6 for each genotype). Asterisks indicate significant differences (*P*<0.05).

To characterize the metabolic activity of the *Bmal1* -/- mice, in a first set of experiments, the respiratory quotient (RQ) was determined. Mice were housed in a chamber connected to indirect calorimeter under a light/dark (12 h/12 h) condition (LD) or constant darkness condition (DD). To obtain RQ, the amount of CO_2_ eliminated from the body and the amount of O_2_ consumed were measured. As shown in Fig. 1, the daily value of the RQ in the *Bmal1* -/- mice was greater than that in control mice under both the LD condition and DD condition.

### High-fat diet increases circulating fatty acids level and ectopic fat accumulation in *Bmal1* -/- mice

**Figure 2:**
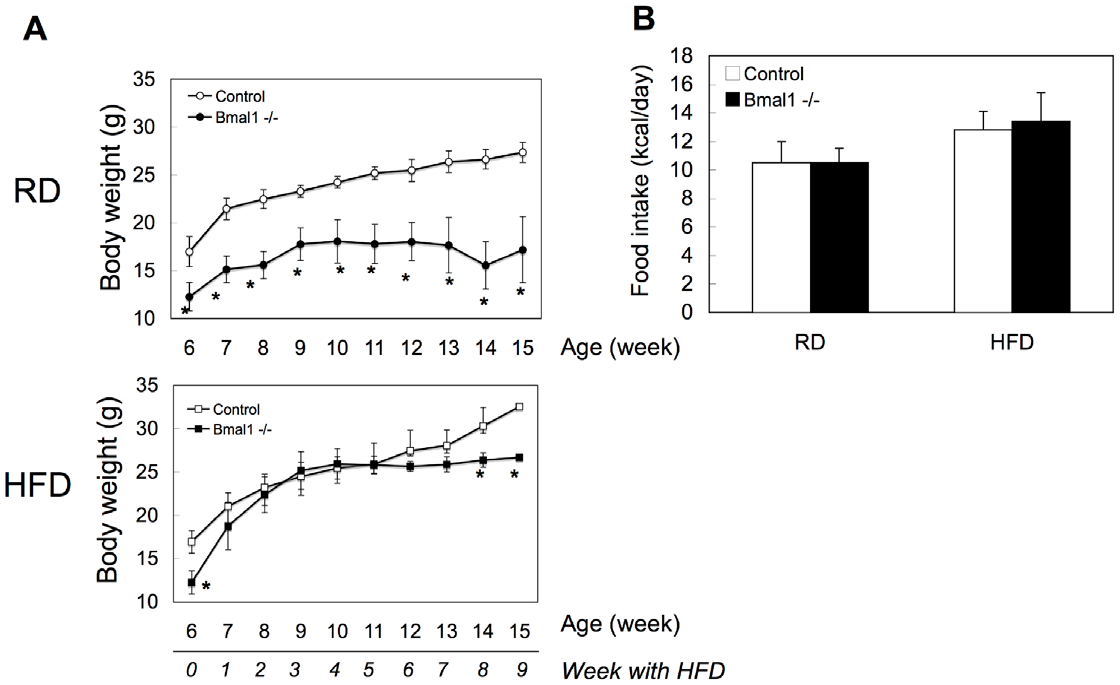
Effects of a high-fat diet feeding on the body weight. (A) Male control mice and *Bmal1* -/- mice were subjected to a high-fat diet (HFD) challenge at 6 weeks of age. The body weights of the mice fed either a regular diet (RD) or HFD were determined. Data represent the means ± SEM (n = 8 for each genotype). Asterisks indicate significant differences (*P*<0.05). (B) The daily average of the calorie intake of male mice at 11 weeks of age (5 weeks with a HFD) was determined. Data represent the means ± SEM (n = 8 for each genotype and treatment).

**Figure 3:**
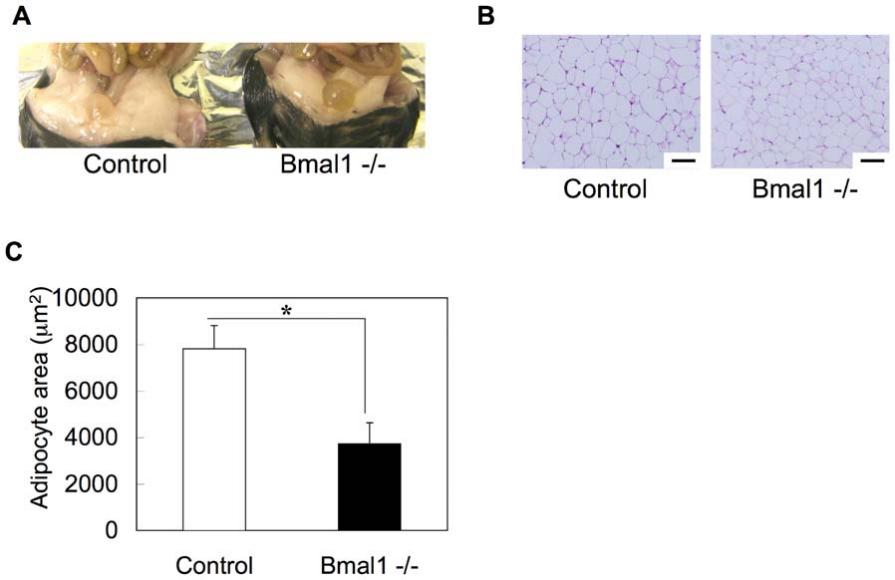
Effects of a high-fat diet feeding on histology of adipocytes in *Bmal1* -/- mice. Adipose tissue was excised at zeitgeber time (ZT) 10. (A) Representative morphology of adipose tissue in control mice and *Bmal1* -/- mice fed a HFD for 5 weeks. (B) Representative HE staining of epididymal white adipose tissue isolated from male control mice and *Bmal1* -/- mice fed a HFD for 5 weeks. Scale bars indicate 100 µm. (C) Average cross-sectional area of individual adipocytes in control mice and *Bmal1* -/- mice fed a HFD. Data represent the means ± SEM (n = 5 for each genotype). Asterisks indicate significant differences (*P*<0.05).

**Figure 4:**
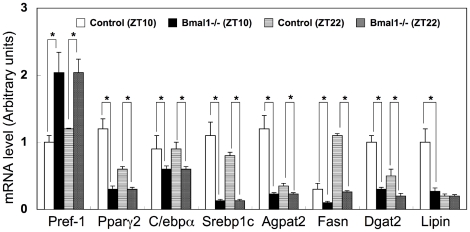
Comparison of gene expressions in adipose tissues. Gene expressions in adipose tissues in control mice and *Bmal1 -/-* mice at ZT10 and 22 were determined by RT-qPCR. Relative mRNA levels were normalized to the 36B4 level. Data represents the means ± SEM (n = 5 for each genotype and point). Asterisks indicate significant differences (*P*<0.05).

The higher RQ value in *Bmal1* -/- mice compared with control mice suggested lower utilization of fat as an energy source in *Bmal1* -/- mice (Fig. 1). To confirm this, *Bmal1* -/- mice were subjected to a high-fat diet challenge. As reported previously [30], the body weight of *Bmal1* -/- mice was lighter than that of control mice under a regular diet condition (Fig. 2A). Feeding with a high-fat diet induced body-weight gain in both control mice and *Bmal1* -/- mice (Fig. 2A). However, while the body weight of control mice continuously increased during the challenge, that of *Bmal1* -/- mice remained almost constant after 4 weeks of the challenge (Fig. 2A). There were no substantial differences in the calorie intake of control mice and *Bmal1* -/- mice during the challenge (Fig. 2B). After 5 weeks of the high-fat diet challenge, the adipose tissue mass in *Bmal1* -/- mice was smaller than that in control mice (Fig. 3A). Also, a histological study showed that the size of adipocytes in *Bmal1* -/- mice was smaller than that in control mice (Fig. 3B and C). As summarized in Fig. 4 and Fig. S4, gene expression of adipogenesis-related transcription factors and a series of factors responsible for synthesis of TG and fatty acids were substantially decreased in adipose tissue in *Bmal1* -/- mice. Furthermore, a significant decrease in the expression of factors involved in fat burning, such as *Ucp3* and *β3 adrenalin receptor*, was observed in *Bmal1* -/- mice (Fig. S4). In contrast, *Pref-1*, a specific marker gene for preadipocytes, was highly expressed in adipose tissue in *Bmal1* -/- mice (Fig. 4).

**Figure 5:**
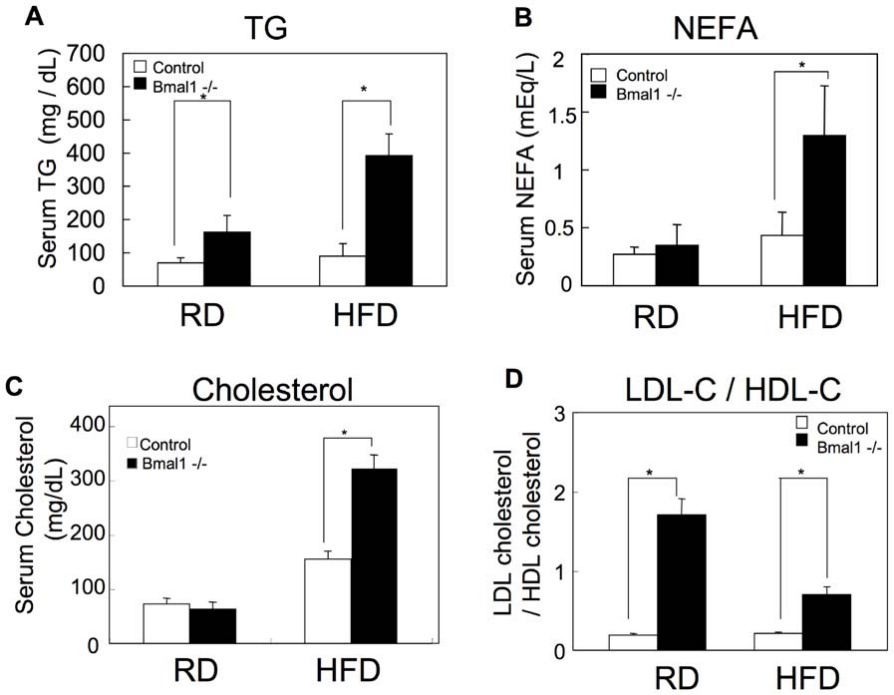
Alteration of circulating fatty acids level in *Bmal1* -/- mice. Serum samples were prepared from male mice fed a regular diet (RD) or a high-fat diet (HFD) for 5 weeks. All samples were collected at ZT 10. (A) Triglycerides (TG). (B) Non-esterified fatty acids (NEFA). (C) Total cholesterol. Data represent the means ± SEM (n = 8 for each genotype and treatment). Asterisks indicate significant differences (*P*<0.05). (D) The LDL cholesterol/HDL cholesterol ratio was calculated based on the data shown in Table 1. Data represent the means ± SEM (n = 8 for each genotype and treatment). Asterisks indicate significant differences (*P*<0.05).

In the next set of experiments, several biochemical markers were determined. The level of serum TG in *Bmal1* -/- mice was higher than that in control mice under the regular-diet feeding condition, and the difference was further pronounced in mice fed a high-fat diet (Fig. 5A). Also, the level of serum NEFA and cholesterol in *Bmal1* -/- mice became higher than that in control mice by feeding a high-fat diet (Fig. 5B and C). Detailed profiling of the cholesterol content in the lipoprotein fractions revealed an increase of the cholesterol level in chylomicron (CM), very low density lipoprotein (VLDL), and low density lipoprotein (LDL) in *Bmal1* -/- mice fed a regular diet (Table 1). Calculation of the TG/cholesterol molar ratio revealed a reduction in the relative content of TG in the lipoprotein complex in *Bmal1* -/- mice (Table 2). The LDL cholesterol/HDL cholesterol ratio, a potent marker for risk of arteriosclerosis, in *Bmal1* -/- mice was remarkably greater than that in control mice (Fig. 5D).

**Table 1:** Content of cholesterol and triglycerides in lipoproteins.

		Cholesterol (mg/dL)				Triglyceride (mg/dL)			
		CM	VLDL	LDL	HDL	CM	VLDL	LDL	HDL
RD	Control	0.1±0.0	2.9±0.2	11.1±0.1	59.4±5.8	0.6±0.1	15.6±1.4	9.5±1.1	1.7±0.2
	Bmal1-/-	0.3±0.0*	7.3±0.8*	20.5±0.3*	35.2±4.8*	4.6±0.5*	19.4±2.1	12.7±1.4*	2.3±0.2*
HFD	Control	0.2±0.0	5.7±0.6	25.9±3.8	123.2±20	3.5±0.4	18.8±1.9	6.5±0.7	1.8±0.1
	Bmal1-/-	0.3±0.0	8.3±1.0*	128.2±14*	184.7±23*	5.3±0.6*	9.1±1.1*	14.9±1.8*	3.4±0.4*

Blood samples were collected at ZT 10. Plasma samples were prepared from male control mice and *Bmal1 -/-* mice fed either a regular diet (RD) or a high fat diet (HFD) for 5 weeks. Plasma lipoproteins were separated and analyzed by an on-line dual enzymatic method for simultaneous quantification of cholesterol and triglycerides by HPLC. Data represent the means ± SEM (n = 5 for each genotype). Asterisks indicate significant differences (*P*<0.05).

**Table 2:** Molar ratio of triglycerides/cholesterol.

		CM	VLDL	LDL	HDL
RD	Control	2.60	2.36	0.38	0.01
	Bmal1-/-	7.76	1.17	0.27	0.03
HFD	Control	7.67	1.45	0.11	0.01
	Bmal1-/-	7.74	0.48	0.05	0.01

Molar ratio was calculated from the data shown in Table 1. Molecular weights of 882.43 and 386.65 were used for triglycerides and cholesterol, respectively.

**Figure 6:**
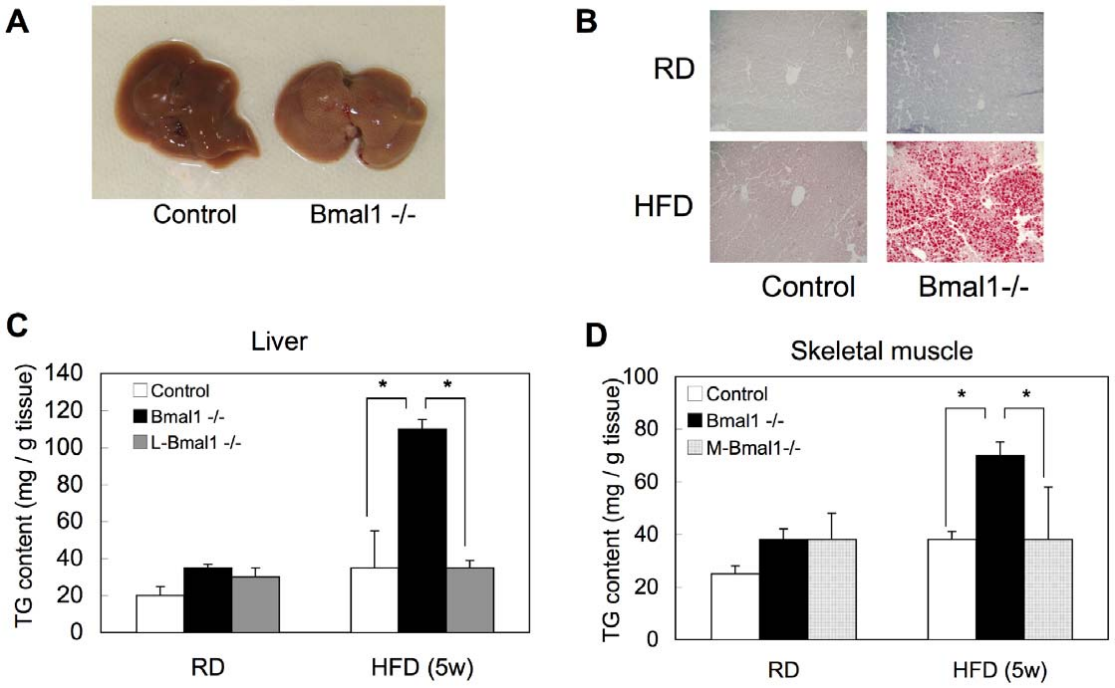
Ectopic fat accumulation in liver and skeletal muscle in *Bmal1* -/- mice. All tissue samples were excised at ZT10. (A) Representative morphology of the livers from control mice and *Bmal1* -/- mice fed a HFD for 5 weeks. (B) Representative oil red O staining of liver tissue samples isolated from male control mice and *Bmal1* -/- mice fed either a RD or HFD for 5 weeks. (C and D) Triglyceride (TG) contents in the liver (C) and skeletal muscle (D) from male control mice and *Bmal1* -/- mice fed a RD or HFD for 5 weeks. Data represent the means ± SEM (n = 8 for each genotype and treatment). Asterisks indicate significant differences (*P*<0.05).

High fat diet feeding induced fatty liver in *Bmal1* -/- mice but not in control mice (Fig. 6A and B). Quantification of tissue TG content revealed significant accumulation of TG in the liver and skeletal muscle in *Bmal1* -/- mice (Fig. 6C and D). Interestingly, under both normal diet and high fat diet conditions, TG contents in the liver in liver-specific (L-) *Bmal1* -/- mice and the skeletal muscle in skeletal muscle-specific (M-) *Bmal1* -/- mice were almost equal to those in control mice (Fig. 6C and D).

### Loss of the *Bmal1* gene alters the expression level of metabolism-related genes

**Figure 7:**
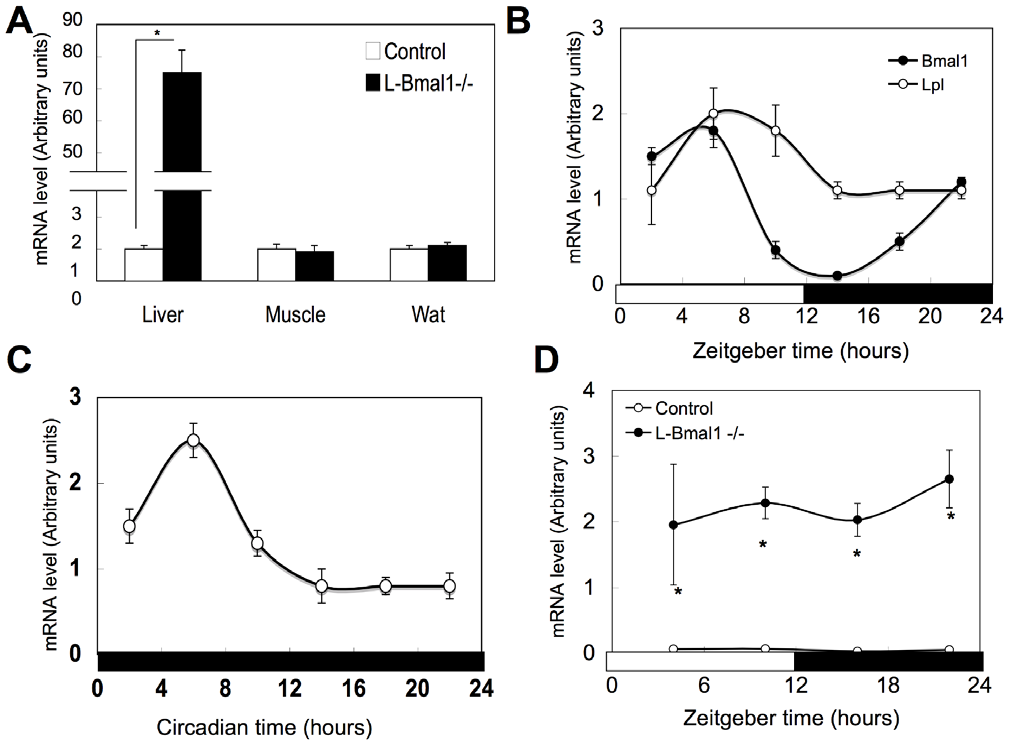
Induction of Lpl gene expression in the liver by the deletion of *Bmal1* gene. (A) Tissue samples were excised form male control mice and L-*Bmal1* -/- mice at ZT10. The total RNA was extracted from these tissues and the expression of Lpl mRNA was determined by RT-qPCR. Relative mRNA levels were normalized to the 36B4 level. Data represent the mean ± SEM (n = 5 for each genotype). Asterisks indicate significant differences (*P*<0.05). Data represent the means ± SEM (n = 5 for each genotype and point). (B) The liver was excised male control mice at the indicated times. The open bar indicates the lights-on phase, and the dark bar indicates lights-off. The total RNA was extracted, and the expression of Bmal1 and Lpl mRNA was determined as described in (A). Data represent the means ± SEM (n = 5 for each point). (C) The liver was excised from the control mice housed in constant darkness for 10 days. The expression of Lpl mRNA was determined as described in (A). Data represent the means ± SEM (n = 5 for each point). (D) The liver was excised male control mice and L-*Bmal1* -/- mice at the indicated times. The open bar indicates the lights-on phase, and the dark bar indicates lights-off. The total RNA was extracted, and the expression of Lpl mRNA was determined as described in (A). Data represent the means ± SEM (n = 5 for each genotype and point). Asterisks indicate significant differences (*P*<0.05).

To gain insight into the ectopic fat accumulation in *Bmal1* -/- mice, gene expression in the liver and muscle was compared to that in control mice by RT-qPCR. Changes in the expression of selected lipid metabolism-related genes are summarized in Table 3 and S1. In the *Bmal1* -/- mice liver, a robust (∼78-fold) increase in *Lpl* expression was observed (Table 3). This increased expression of *Lpl* gene was also observed in the liver in L-*Bmal1* -/- mice (Fig. 7A and D) and in the *Bmal1* knockdown cultured hepatocytes prepared from control mice (Fig. S5). The expression pattern of *Lpl* showed clear circadian oscillations in the liver of control mice under both the LD and DD condition (Fig. 7B–D). The expression of genes involved in fatty acid binding and transport, namely *Cd36*, *Fabp5*, and *Srbi*, were induced in the *Bmal1* -/- mice liver (Table 3). In the skeletal muscle, the expression of genes in the pathway for *de novo* fatty acid synthesis such as *Fasn* and *Scd1*, and *Agpat3* were highly induced (Table 3). Conversely, *Ldlr* expression was significantly decreased in *Bmal1* -/- skeletal muscle (Table 3). Expression of the *Lpl* gene in *Bmal1* -/- skeletal muscle was not significantly different compared to that in control mice (Table 3). In both liver and muscle, expression of genes responsible for oxidation of fatty acids was decreased (Table 3).

**Table 3:** Gene expressions in *Bmal1* -/- mice.

	Liver		Muscle	
	ZT10	ZT22	ZT10	ZT22
**Fatty acid binding and transpot**				
Cd36	2.7±0.3*	1.1±0.1	1.2±0.2	1.4±0.2
Fabp5	4.3±0.4*	1.5±0.1*	1.1±0.1	0.9±0.2
Ldlr	0.8±0.3	1.0±0.2	0.2±0.1*	0.2±0.2*
Lpl	77.9±8.5*	79.3±9.1*	1.1±0.2	0.9±0.1
Srbi	2.7±0.3*	1.5±0.1*	0.7±0.3	1.1±0.2
**TG and fatty acid synthesis**				
Agat2	0.8±0.8	0.6±0.3	1.1±0.1	1.3±0.2
Agat3	1.0±0.2	1.1±0.1	2.0±0.2*	1.8±0.2*
Dgat1	0.6±0.2*	0.7±0.1	0.9±0.2	1.1±0.1
Dgat2	0.5±0.1*	0.5±0.1*	0.6±0.2*	0.4±0.2*
Fasn	0.3±0.0*	0.3±0.0*	6.3±0.6*	1.1±0.2
Scd1	1.3±0.2	0.3±0.1*	5.0±1.1*	2.0±0.3*
**Fatty acid oxidation**				
Acaa 1a	0.6±0.1*	0.5±0.1*	0.8±0.2	0.7±0.2
Acaa 1b	0.3±0.1*	0.2±0.0*	0.6±0.3	0.6±0.3
Acadl	1.7±0.2	0.7±0.2	1.6±0.2*	0.9±0.2
Acadm	0.7±0.3	0.7±0.2	1.7±0.3*	1.3±0.1
Echdc1	0.6±0.1*	0.5±0.1*	1.3±0.3	0.9±0.1

Gene expressions in control mice and *Bmal1 -/-* mice were determined by RT-qPCR. Relative mRNA levels were normalized to the 36B4 level. Each value represented the mean fold-changes (± SEM) of *Bmal1 -/-* mice in comparison to control mice (n = 5 for each genotype and point). Asterisks indicate significant differences (*P*<0.05).

## Discussion

A link between circadian rhythm and metabolism has long been discussed [31], [32]. Therefore, in this study, we aimed to study whether loss of BMAL1 functions induces metabolic syndrome. Deletion of the *Bmal1* gene resulted in an elevation of the daily average value of the RQ under both the LD and DD conditions (Fig. 1). An increase in the RQ represents oxidation of carbohydrates, and a decrease of the value represents oxidation of fatty acids. Thus, the elevation of RQ in *Bmal1* -/- mice indicates that BMAL1 is involved in the utilization of fat as an energy source. Indeed, under the high-fat diet challenge, the levels of circulating fatty acids, including TG, NEFA and cholesterol, in *Bmal1* -/- mice were remarkably increased (Fig. 5A–C). Elevation of circulating fatty acids induced ectopic fat accumulation in the liver and muscle (Fig. 6A–D). A detailed profile analysis of the cholesterol contents in lipoprotein fractions revealed that the ratio of LDL cholesterol to HDL cholesterol, a potent marker for risk of arteriosclerosis, in *Bmal1* -/- mice was significantly higher than that in control mice even under a regular-diet condition (Fig. 5D). Furthermore, Hemmeryckx et al. recently reported endothelial dysfunction and progression of the prothrombotic state in *Bmal1 -/-* mice [26]. Consequently, we were led to conclude that the loss of the *Bmal1* gene induced metabolic syndrome.

**Figure 8:**
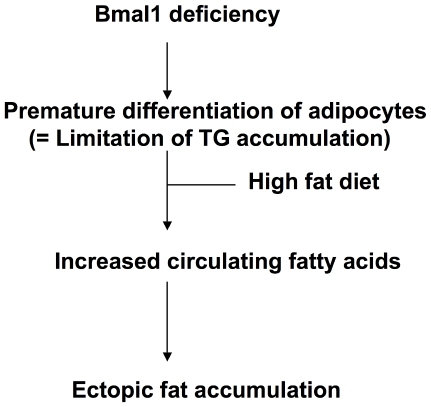
A schematic diagram of Bmal1 deficiency-induced abnormal lipid metabolism.

Ectopic fat formation by high fat feeding was not observed in L-*Bma1* -/- mice and M-*Bmal1* -/- mice, suggesting that elevation of the level of circulating fatty acids level was a main cause of ectopic fat formation in the tissues. As described above, the RQ value in *Bmal1* -/- mice is higher than that in control mice (Fig. 1). Also, excess secretion of an oily, waxy substance (sebum) was observed in *Bmal1* -/- mice fed a high-fat diet (Fig. S6). These results suggest that the ability to utilize and store fat in *Bmal1* -/- mice is inferior to that in control mice. Excess fatty acids are largely deposited in the adipose tissue. Histological analysis showed that the size of adipocytes in *Bmal1* -/- mice fed a high-fat diet was significantly smaller than that in control mice (Fig. 3A–C). Since expression of *Ucp3* and *β3 adrenalin receptor* was down-regulated in the *Bmal1* -/- mice, excess of fatty acid oxidation was unlikely the cause of decrease of adipocyte size (Fig. S4). As we previously demonstrated, BMAL1 plays central roles in the regulation of high fat diet-induced adipogenesis [22]. Indeed, the expression level of the *Pref-1* gene, a marker for preadipocytes, in *Bmal1* -/- mice adipose tissue was greater than that in control mice (Fig. 4). In contrast, the genes preferentially expressed in mature adipocytes, such as *Pparγ2*, *C/ebpα*, and *Srebp1c*, were expressed at lower levels in *Bmal1* -/- mice (Fig. 4). Furthermore, the expressions of *Agpat 2* and *lipin* were significantly reduced in *Bmal1* -/- mice adipose tissue (Fig. 4). Mutations in *Agpat 2* and *lipin* cause congenital generalized lipodystrophy [33], [34]. As has been demonstrated in lipodystrophy, the failure of adipose tissue mass to expand and accommodate a high-energy influx causes accumulation of ectopic fat [35], [36]. Therefore, we are led to conclude that an increase of the circulating fatty acids level followed by the ectopic fat accumulation in the *Bmal1* -/- mice was mainly due to the insufficient function of adipose tissue resulting from lower adipogenesis activity (Fig. 8).

Although both *Bmal1* -/- mice and *Clock* mutant mice develop metabolic syndrome-like symptoms, *Bmal1* -/- mice are thin and *Clock* mutant mice are obese (28, 29, this study). As mentioned above, metabolic disorder in *Bmal1* -/- mice was induced as a result of lower adipogenesis activity, as observed in lipodystrophy. On the other hand, *Clock* mutant mice develop metabolic syndrome-like symptoms presumably as a result of obesity. In *Clock* mutant mice, expression level of *Bmal1* is significantly increased [37]. Therefore, one possibility is that BMAL1 overexpressed alone or in cooperation with other genes, such as Npas2, promotes adipogenesis. Alternatively, BMAL1/mutated CLOCK complex may act as a dominant negative factor and affect several gene expression involved in regulation of lipid metabolism.

Recently, Marcheva et al. reported that BMAL1 and CLOCK play roles in the regulation of pancreas functions, including the proliferation of islets and secretion of insulin in response to glucose [25]. Impaired insulin secretion and glucose intolerance in *Bmal1-/-* mice were also observed in this study, as summarized in Text S1, Fig. S7 and S8. Therefore, one can speculate that the elevation of circulating fatty acids is partly due to impaired insulin secretion in *Bmal1* -/- mice. Under the condition of the impaired insulin actions, phosphorylation of Ser residues at 563 and 660 in hormone sensitive lipase (HSL) in adipose tissue is accelerated, resulting in increase of lipolysis [38]. In *Bmal1-/-* mice, despite the lower insulin secretion, the phosphorylation status of Ser563 and 660 was substantially lower than that in control mice (Fig. S9). Therefore, although we do not exclude the possibility that, in *Bmal1* -/- mice, impaired insulin secretion is partly responsible for development of dyslipidemia, its contribution via acceleration of lipolysis is relatively smaller.

Lack of *Bmal1* induced a robust induction of *Lpl* expression in the liver (Table 3). A detailed analysis of the lipoprotein complex revealed that VLDL and LDL in *Bmal1* -/- mice were relatively TG poor (Table 2), suggesting that LPL activity is substantially increased in *Bmal1* -/- mice. This strong induction of *Lpl* gene expression was observed in the liver of L-*Bmal1* -/- mice, but not in other tissues, and *Bmal1*-knockdown cultured hepatocytes (Fig. 7A and D, Table 3, and Fig. S5). These results indicate that overexpression of the hepatic *Lpl* gene was mainly due to the lack of BMAL1 functions in the liver. Previous studies using liver-specific *Lpl* transgenic mice demonstrated that overexpression of the *Lpl* gene resulted in accumulation of TG in the liver even under a regular-diet condition [39]. Loss of the *Bmal1* gene also induced *Cd36*, *Fabbp5*, and *Srbi* in the liver (Table 3). Consequently, ectopic fat formation in the liver may be induced by increased fatty acid transport under the condition of elevated levels of circulating fatty acids.

Overall, this study showed that BMAL1 activity is closely associated with lipid metabolism. It is known that several factors regulating metabolic activity affect *Bmal1* functions. One of the well-characterized examples is the redox state of NAD [40]. The reduced forms of the redox cofactors, NAD (H) and NADP (H), strongly enhanced DNA binding of the BMAL1/CLOCK, whereas the oxidized forms inhibited it [40]. Not only is NAD important in cellular redox reactions and the DNA-binding activity of BMAL1/CLOCK, as described above, but it also serves as a substrate for sirtuin (SIRT) 1, an NAD-dependent and nutrient-responsive deacetylase [41], [42]. SIRT1 activated with NAD reduces the acetylation status of BMAL1, resulting in the suppression of BMAL1/CLOCK activity [43]. In diabetic rats lacking insulin, the phase of the circadian gene expression in the heart is advanced, suggesting that high blood glucose levels can phase-shift the clock in peripheral tissues [44]. It has been shown that ethanol consumption alters the circadian expression pattern of *Per* genes [45]. Although it is unknown how ethanol affects the circadian clock, it may be possible that NADH generated by alcohol metabolism affects the activity of BMAL1/CLOCK.

In conclusion, the results in this study revealed that loss of the functions of BMAL1 leads to the development of ectopic fat formation and dyslipidemia. Therefore, the present results may provide an excellent opportunity to gain new insights into the regulation of energy homeostasis by BMAL1.

## Materials and Methods

### Animals

Conditional *Bmal1* (flox/flox) mice, which carry the conditional *Bmal1* allele containing exons 6 to 8 flanked by loxP sites, were generated using ES cells derived from C57BL/6J mice. Splicing of exons 6 to 8 should cause a deletion of the bHLH domain and a frameshift mutation with the introduction of an early stop codon (TGA). Global (whole body) *Bmal1* deficient (-/-) mice were generated by breeding of *Bmal1* (flox/flox) mice with knockin C57BL/6J mice carrying the Cre recombinase gene driven by a PGK promoter (Ozgene, Perth, Australia). Genotyping for exons 6–8 was performed by PCR on DNA isolated from tail biopsies by using the forward primer A (5′- GGGGATTTCCATCTGTGTTTAC-3′) and primer B (5′- CTCATCTGCTTATCTGCTCTGGGG -3′). The *Bmal1*-excised allele amplified a 280-bp band, whereas amplification from the *Bmal1*-unexcised allele resulted in a 2500-bp band. Analysis of the excision of exon 8 was carried out by PCR using primer C (5′-CCTGGAACTCACTTTGTAGACC-3′) and primer D (5′- AACAGCCATCCTTAGCACG-3′). A 250-bp band confirmed the presence of a gene corresponding to exon 8. To obtain mice harboring liver or skeletal muscle with excision at *Bmal1*, mice expressing the *Bmal1* (flox/flox) allele were crossed to mice expressing a *Cre* transgene driven by either the albumin (Alb) or the muscle creatine kinase (Mck) promoter (Jackson Laboratories, Bar Harbor, ME). Mice homozygous for the floxed allele and hemizygous for the *Cre* transgene (*Bmal1* (flox/flox/Cre^Alb^) or *Bmal1* (flox/flox/Cre^Mck^)) were obtained by crossing *Bmal1* (flox/+/Cre^Alb^) or *Bmal1* (flox/+/Cre^Mck^) mice to *Bmal1* (flox/flox) mice. Littermates that were negative for the *Cre* transgenes (*Bmal1* (flox/flox)) were used as experimental controls. All mice were maintained at 23±1°C with 50±10% relative humidity under a 12 h light/12 h dark cycle (light: zeitgeber time (ZT) 0–12; dark; ZT12-24). For the experiments under constant darkness (DD), mice were entrained to DD condition for 7 days. All procedures during the dark period were conducted using infrared vision and dim red lighting. Food and water were available ad libitum. The high-fat diet consisted of 58% lard (wt/wt), 30% fish powder, 10% skim milk, and a 2% vitamin and mineral mixture (equivalent to 7.5% carbohydrate, 24.5% protein, and 60% fat) (OYC, Tokyo, Japan). The regular diet proportions were 54% carbohydrate, 20% protein, and 4.5% fat (OYC). The experimental protocol was approved by the Ethics Review Committee for Animal Experimentation of Nihon University (Permit number:AP10P006).

### Metabolic studies

Locomotor activity was measured by using an infrared passive sensor system (Muromachi, Tokyo, Japan). Energy expenditure was measured by an indirect calorimeter (Muromachi). For oral glucose tolerance testing (OGTT), dextrose solution (2 g/kg body weight) was administered by oral gavage. Glucose levels were monitored before and at 15, 30, 60, and 120 min postgavage using blood glucose strips (Arkray, Kyoto, Japan). Plasma insulin levels during OGTT were measured by enzyme-linked immunosorbent assay (MIoBS, Yokohama, Japan). Insulin tolerance tests were performed by injecting 0.5 U insulin (Eli Lilly, Indianapolis, IN)/kg body weight intraperitoneally, followed by blood collection at 0, 30, 60, 120, and 180 min after injection. Blood glucose values were determined as described above.

### Biochemical analysis of blood and tissue

Plasma lipoproteins were analyzed by an on-line dual enzymatic method for simultaneous quantification of cholesterol and triglycerides by HPLC at Skylight Biotech Inc. (Akita, Japan) according to the procedure described by Usui et al. [46]. The levels of serum TG, non-esterified fatty acid (NEFA), and total cholesterol were determined using a commercially available reagent (Wako, Tokyo, Japan). Hepatic and muscular lipids were extracted according to the methods of Folch et al. [47]. The extract was dissolved in 2-propanol and subsequently analyzed for TG as described above.

### Analysis of gene expression

Total RNA was extracted using TRIzol reagent (Invitrogen, Carlsbad, CA) according to the manufacturer's instructions. To perform RT-qPCR, total RNA (1 µg) was reverse-transcribed using oligo dT primers. A portion of the cDNA (corresponding to 0.04 µg of total RNA) was amplified on an MX3000P real-time PCR system (Stratagene, La Jolla, CA) using Platinum SYBR Green qPCR SuperMix-UDG (Invitrogen). Information on the sequences of primers is available upon request. Standard curves (R2>0.99) were generated using a serial dilution of cDNA, and expression of all genes was normalized to the 36B4 level.

## Supporting Information

Figure S1 **Schematic diagram of the targeting construct and the resulting mutant allele.** Dotted lines represent the fragment sizes generated by PCR genotyping of control and mutant alleles.
(TIF)

Figure S2 **Genotyping of** ***Bmal1*** **-/- mice.** PCR genotyping of tail biopsies showing bands of 2500 bp and 280 bp indicating the presence of the control and mutant alleles, respectively (Top). The absence of 250 bp indicates deletion of exon 8 (bottom).
(TIF)

Figure S3 **Representative activity records of individual control mice and** ***Bmal1*** **-/- mice.** Animals were individually housed under a light–dark (LD) cycle or constant darkness (DD). The activity was measured by using an infrared passive sensor system (Muromachi, Tokyo, Japan).
(TIF)

Figure S4 **Comparison of gene expressions in adipose tissues.** Gene expressions in adipose tissues in control mice and *Bmal1 -/-* mice at ZT10 and 22 were determined by RT-qPCR. Relative mRNA levels were normalized to the 36B4 level. Data represents the means ± SEM (n = 5 for each genotype and point). Asterisks indicate significant differences (*P*<0.05).
(TIF)

Figure S5 **Expression of** ***Lpl*** **mRNA in** ***Bmal1*** **-knockdown cultured hepatocytes.** Primary hepatocytes were prepared from control mice and treated with siRNA solution (control and Bmal1, final concentration 100 nM each) for 48 h. The total RNA was extracted from these cells, and the gene expression was determined as described in Fig. 7A (n = 4 for each treatment).
(TIF)

Figure S6 **Representative images of control mice and** ***Bmal1*** **-/- mice fed a high-fat diet.** Control mice and *Bmal1* -/- mice were subjected to high-fat diet challenge for 3 days. Excess sebum was observed only in *Bmal1* -/- mice.
(TIF)

Figure S7 **Impaired insulin in** ***Bmal1*** **-/- mice.** All experiments were performed at ZT10. (A, B) Male control mice and *Bmal1* -/- mice were fasted for 16 h. After oral glucose administration (2 g/kg), the levels of blood glucose (A) and plasma insulin (B) were monitored. Data represent the means ± SEM (n = 8 for each genotype and point). Asterisks indicate significant differences (*P*<0.05). (C) Male control mice and *Bmal1* -/- mice were fasted for 6 h. After i. p. injection of insulin (0.5 U/kg), blood glucose levels were monitored. Data represent the means ± SEM (n = 8 for each genotype and point). Asterisks indicate significant differences (*P*<0.05). (D) Blood glucose levels in male control mice and *Bmal1* -/- mice fed ad libitum or fasted for 16 h are shown. Data represent the means ± SEM (n = 8 for each genotype and treatment). (E) The pancreas was isolated from male control mice and *Bmal1* -/- mice. Insulin contents in the tissues were determined by ELISA. The values were corrected by the amount of protein in the tissue. Data represent the means ± SEM (n = 8 for each genotype and point). Asterisks indicate significant differences (*P*<0.05). (F) Response of male control mice and *Bmal1* -/- mice to i.p. injection of glibenclamide (2.5 mg/kg). Data represent the means ± SEM (n = 8 for each genotype and point). Asterisks indicate significant differences (*P*<0.05). (G) Representative morphology of islets stained with anti-insulin antibody. Scale bars indicate 100 µm. (H) The average cross-sectional area of the islets in *Bmal1* -/- mice was compared with that in control mice. Data represent the means ± SEM (n = 4 for each genotype and point). Asterisks indicate significant differences (*P*<0.05).
(TIF)

Figure S8 **Lowered gluconeogenesis activity in the** ***Bmal1*** **-/- mice liver.** All experiments were performed at ZT10. (A) Male control mice and *Bmal1* -/- mice were fasted for 16 h. After i.p. injection of pyruvate (2 g/kg), blood glucose levels were monitored. Data represent the means ± SEM (n = 8 for each genotype and point). Asterisks indicate significant differences (*P*<0.05). Gene expression levels in the liver of male control and *Bmal1* -/- mice were determined by RT-qPCR. Relative mRNA levels were normalized to the 36B4 level. Data represent the means ± SEM (n = 5 for each genotype). Asterisks indicate significant differences (*P*<0.05). Levels of G6P and AMP in the liver in male control and *Bmal1* -/- mice were determined by capillary electrophoresis time-of-flight mass spectrometry. Data represent the means ± SEM (n = 4 for each genotype). Asterisks indicate significant differences (*P*<0.05).
(TIF)

Figure S9 **Phosphorylation status of HSL in** ***Bmal1*** **-/- mice adipose tissue.** Total HSL protein level and phosphorylated form of HSL protein in the adipose tissue isolated from male control mice and Bmal1 -/- mice at ZT10 were determined by Western blot.
(TIF)

Table S1 **Gene expressions in** ***Bmal1*** **-/- mice.** Gene expressions in control mice and *Bmal1 -/-* mice were determined by RT-qPCR. Relative mRNA levels were normalized to the 36B4level. N.D. not determined. Each value represented the mean fold-changes (± SEM) of *Bmal1 -/-* mice in comparison to control mice (n = 5 for each genotype and point). Asterisks indicate significant differences (*P*<0.05).
(DOC)

Text S1 **Impaired glucose deposition and acceleration of gluconeogenesis in** ***Bmal1*** **-/- mice.**
(DOC)
