# Supplementary figures and images for: Deficient of a Clock Gene, Brain and Muscle Arnt-Like Protein-1 (BMAL1), Induces Dyslipidemia and Ectopic Fat Formation
Source: PLoS One. 2011 Sep 22;6(9):e25231. doi: 10.1371/journal.pone.0025231 (PMC3178629; doi:10.1371/journal.pone.0025231)

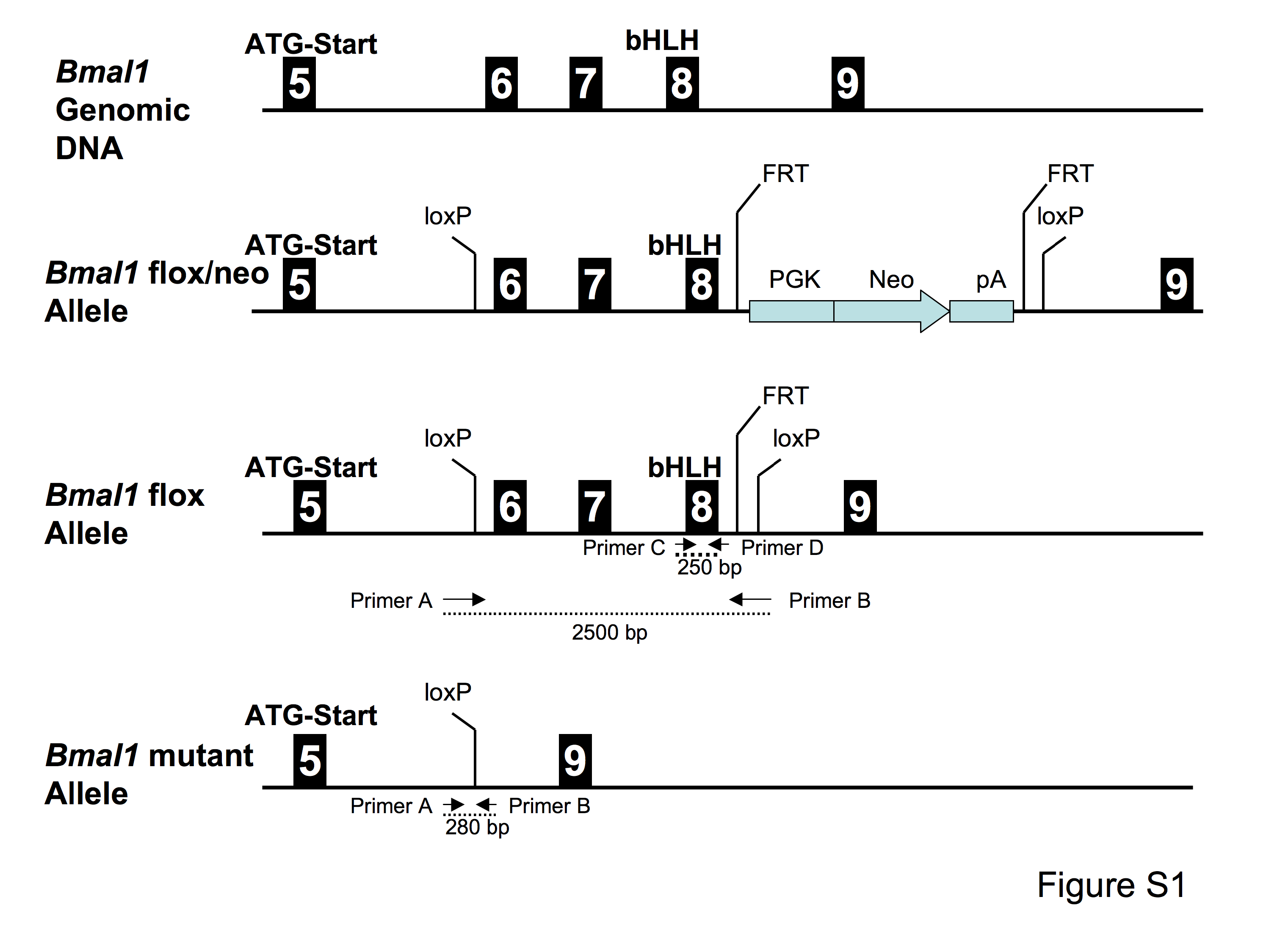

Supplement: Figure S1 — Schematic diagram of the targeting construct and the resulting mutant allele. Dotted lines represent the fragment sizes generated by PCR genotyping of control and mutant alleles. (TIF) [file pone.0025231.s001.tif]

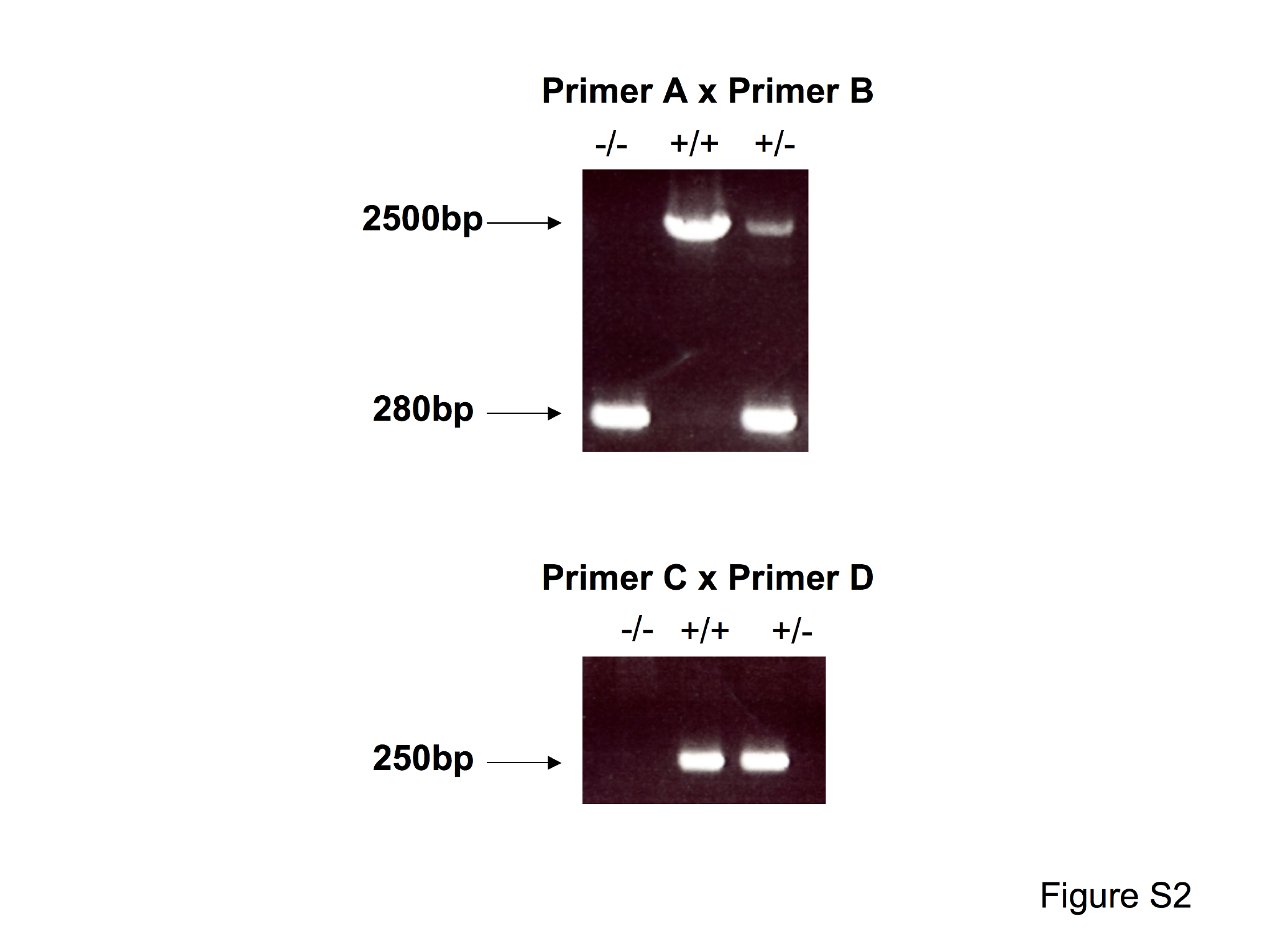

Supplement: Figure S2 — Genotyping of Bmal1 -/- mice. PCR genotyping of tail biopsies showing bands of 2500 bp and 280 bp indicating the presence of the control and mutant alleles, respectively (Top). The absence of 250 bp indicates deletion of exon 8 (bottom). (TIF) [file pone.0025231.s002.tif]

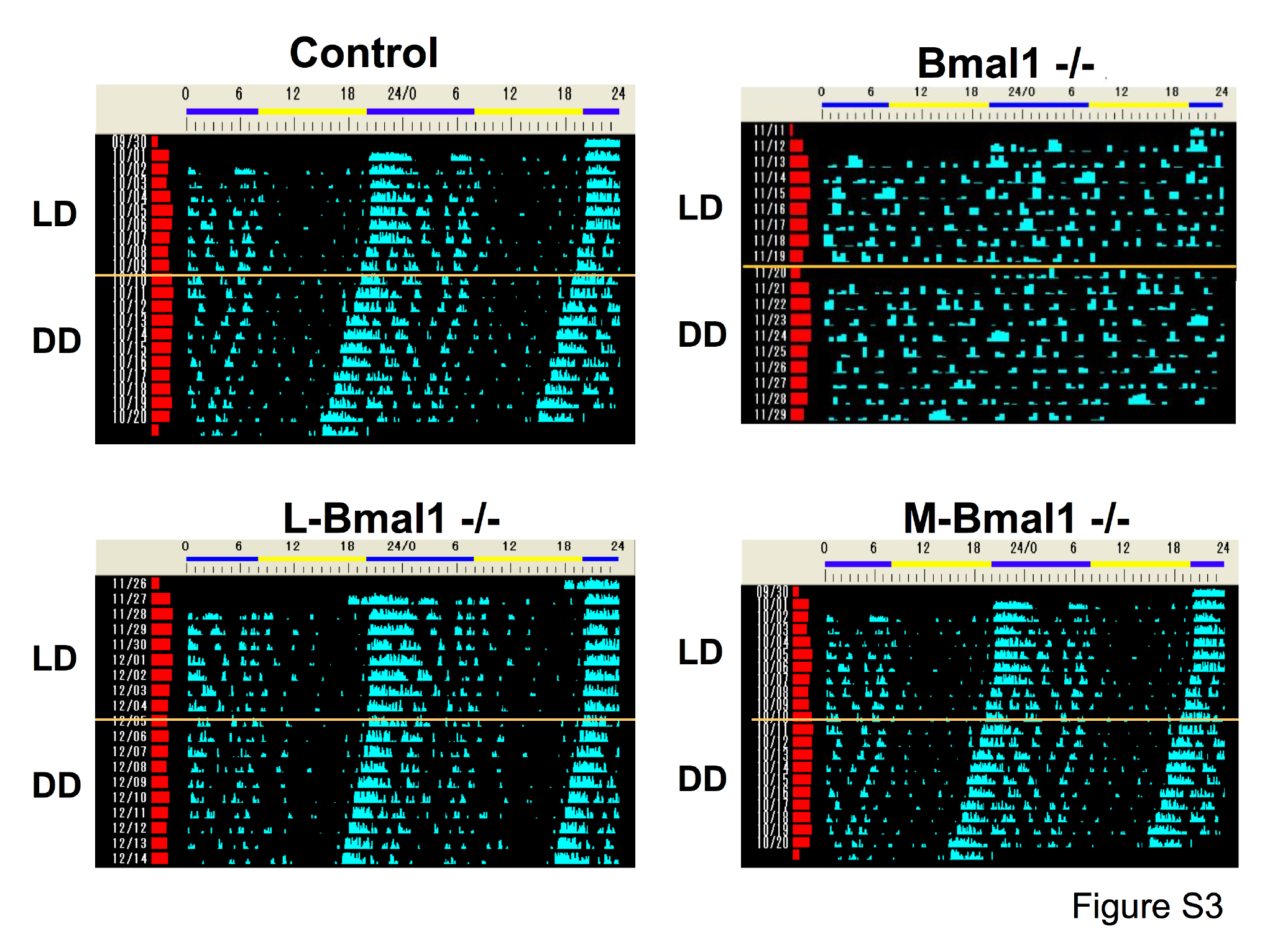

Supplement: Figure S3 — Representative activity records of individual control mice and Bmal1 -/- mice. Animals were individually housed under a light–dark (LD) cycle or constant darkness (DD). The activity was measured by using an infrared passive sensor system (Muromachi, Tokyo, Japan). (TIF) [file pone.0025231.s003.tif]

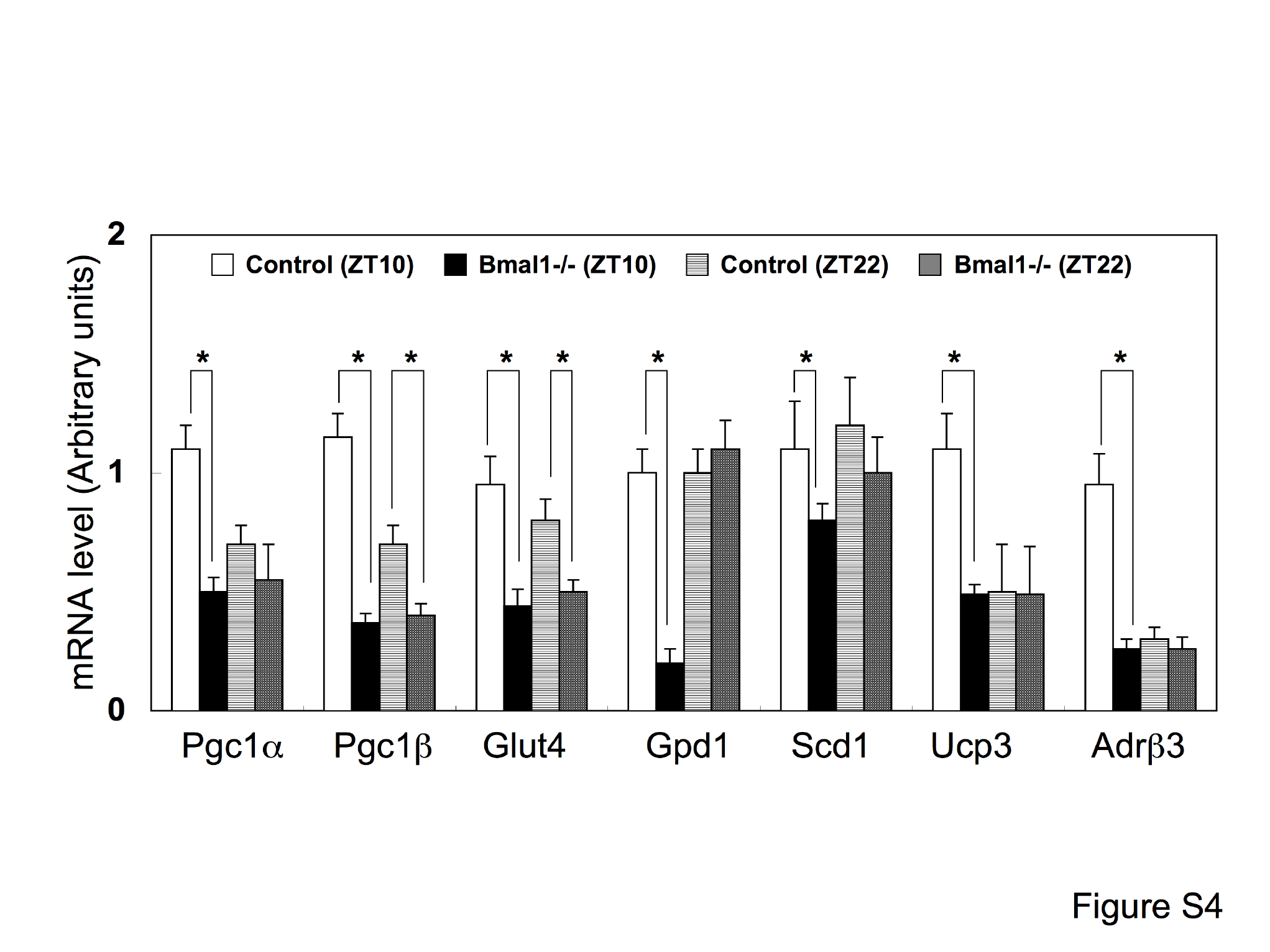

Supplement: Figure S4 — Comparison of gene expressions in adipose tissues. Gene expressions in adipose tissues in control mice and Bmal1 -/- mice at ZT10 and 22 were determined by RT-qPCR. Relative mRNA levels were normalized to the 36B4 level. Data represents the means ± SEM (n = 5 for each genotype and point). Asterisks indicate significant differences (P<0.05). (TIF) [file pone.0025231.s004.tif]

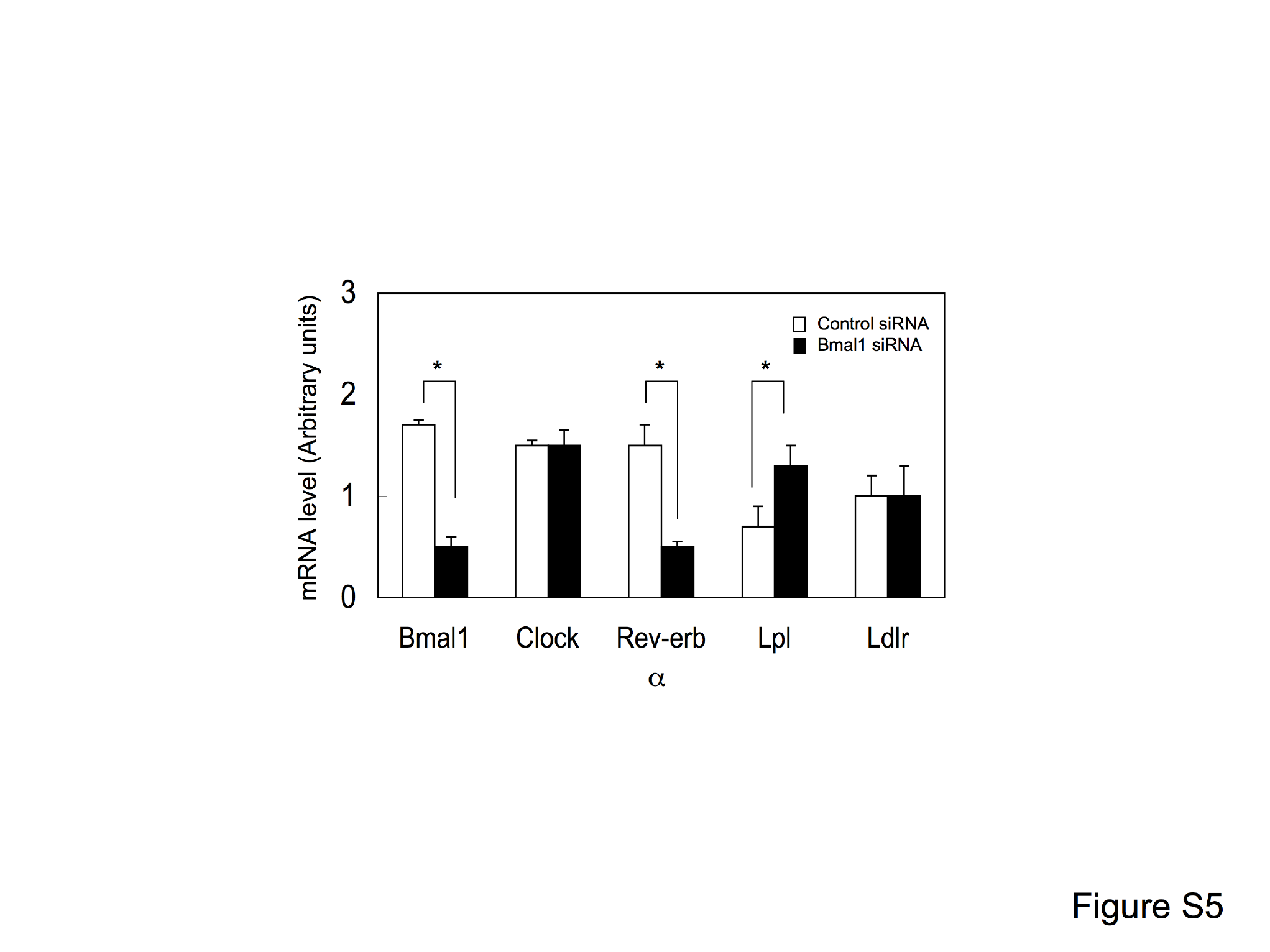

Supplement: Figure S5 — Expression of Lpl mRNA in Bmal1 -knockdown cultured hepatocytes. Primary hepatocytes were prepared from control mice and treated with siRNA solution (control and Bmal1, final concentration 100 nM each) for 48 h. The total RNA was extracted from these cells, and the gene expression was determined as described in Fig. 7A (n = 4 for each treatment). (TIF) [file pone.0025231.s005.tif]

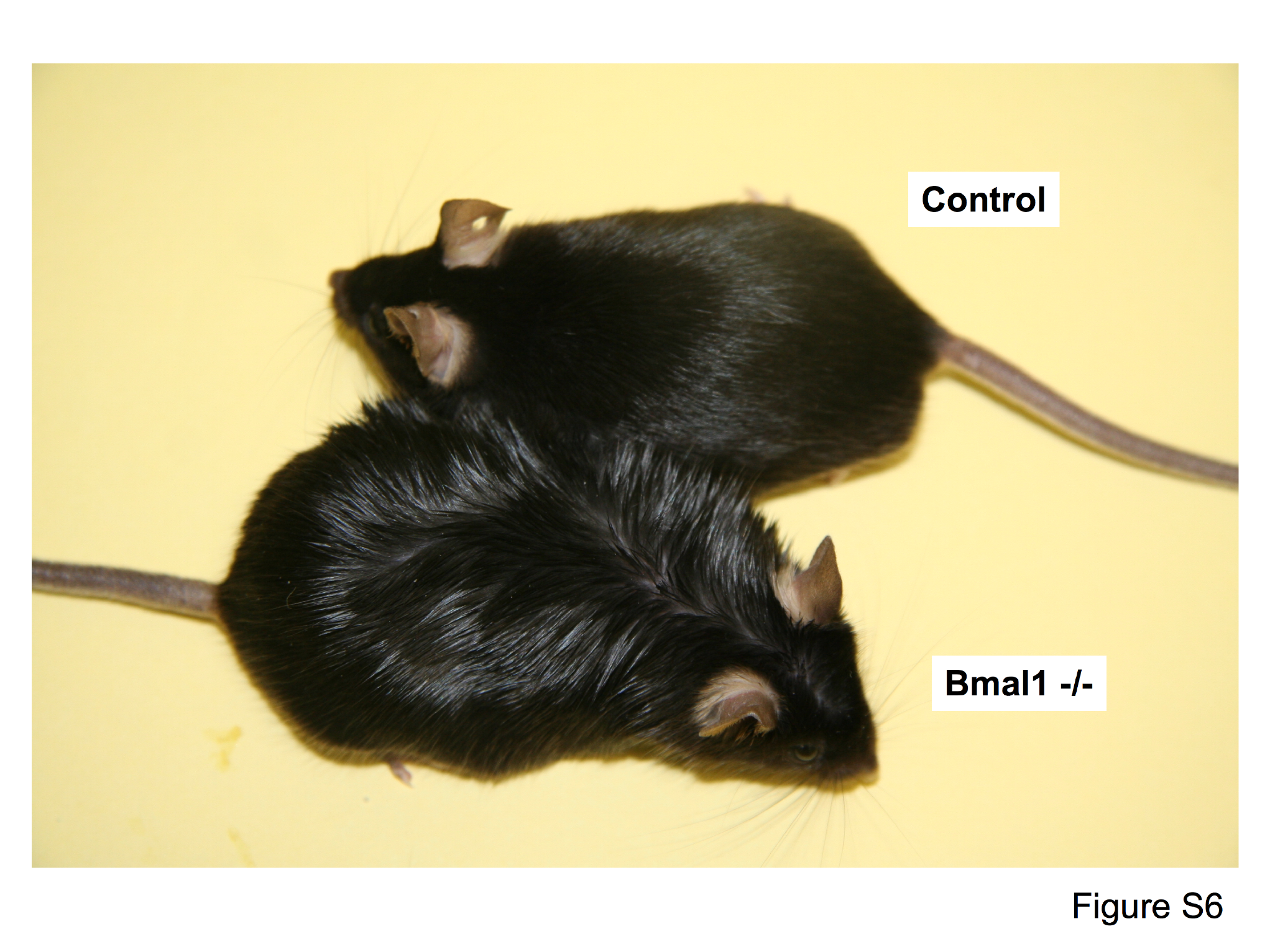

Supplement: Figure S6 — Representative images of control mice and Bmal1 -/- mice fed a high-fat diet. Control mice and Bmal1 -/- mice were subjected to high-fat diet challenge for 3 days. Excess sebum was observed only in Bmal1 -/- mice. (TIF) [file pone.0025231.s006.tif]

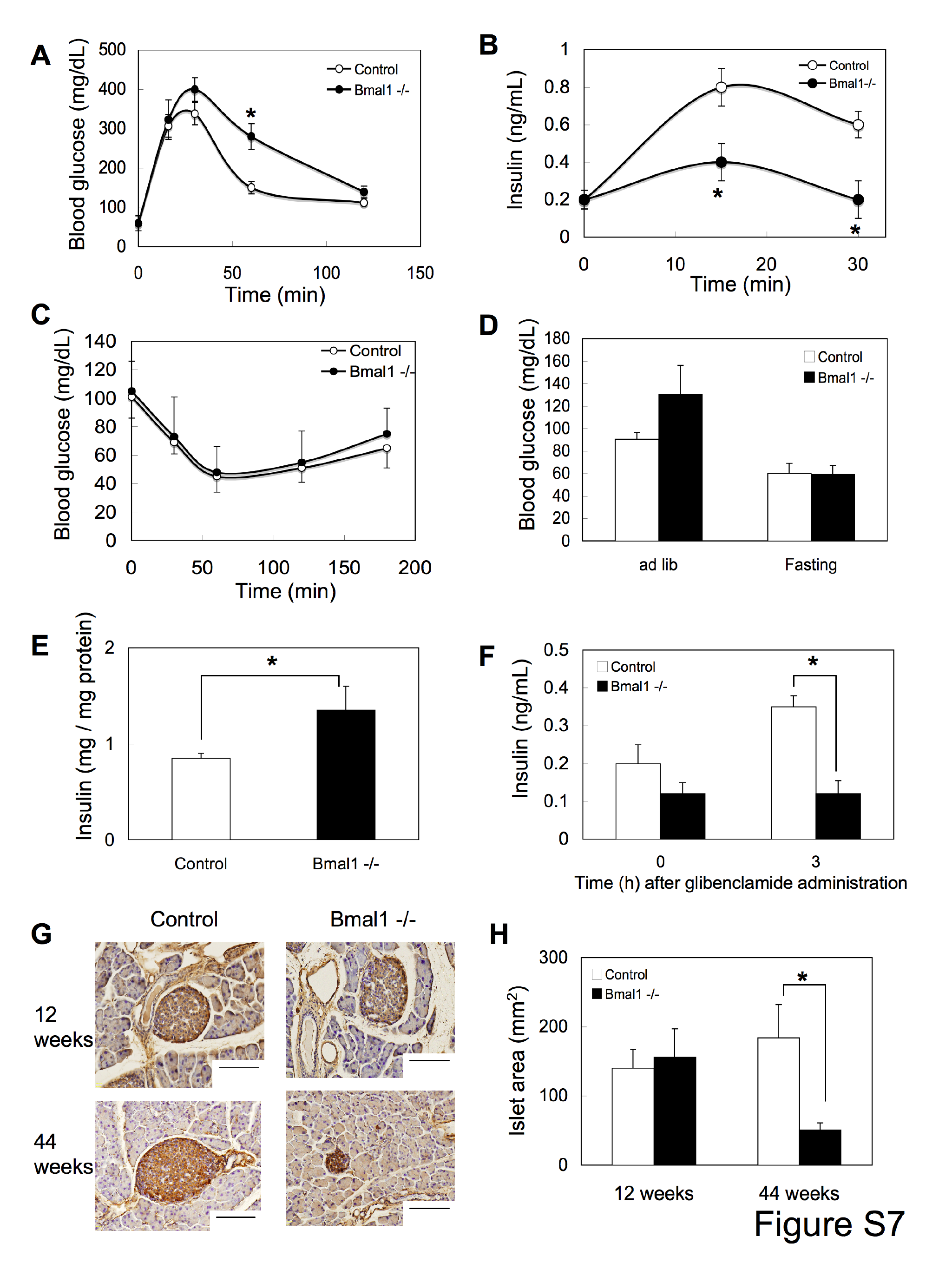

Supplement: Figure S7 — Impaired insulin in Bmal1 -/- mice. All experiments were performed at ZT10. (A, B) Male control mice and Bmal1 -/- mice were fasted for 16 h. After oral glucose administration (2 g/kg), the levels of blood glucose (A) and plasma insulin (B) were monitored. Data represent the means ± SEM (n = 8 for each genotype and point). Asterisks indicate significant differences (P<0.05). (C) Male control mice and Bmal1 -/- mice were fasted for 6 h. After i. p. injection of insulin (0.5 U/kg), blood glucose levels were monitored. Data represent the means ± SEM (n = 8 for each genotype and point). Asterisks indicate significant differences (P<0.05). (D) Blood glucose levels in male control mice and Bmal1 -/- mice fed ad libitum or fasted for 16 h are shown. Data represent the means ± SEM (n = 8 for each genotype and treatment). (E) The pancreas was isolated from male control mice and Bmal1 -/- mice. Insulin contents in the tissues were determined by ELISA. The values were corrected by the amount of protein in the tissue. Data represent the means ± SEM (n = 8 for each genotype and point). Asterisks indicate significant differences (P<0.05). (F) Response of male control mice and Bmal1 -/- mice to i.p. injection of glibenclamide (2.5 mg/kg). Data represent the means ± SEM (n = 8 for each genotype and point). Asterisks indicate significant differences (P<0.05). (G) Representative morphology of islets stained with anti-insulin antibody. Scale bars indicate 100 µm. (H) The average cross-sectional area of the islets in Bmal1 -/- mice was compared with that in control mice. Data represent the means ± SEM (n = 4 for each genotype and point). Asterisks indicate significant differences (P<0.05). (TIF) [file pone.0025231.s007.tif]

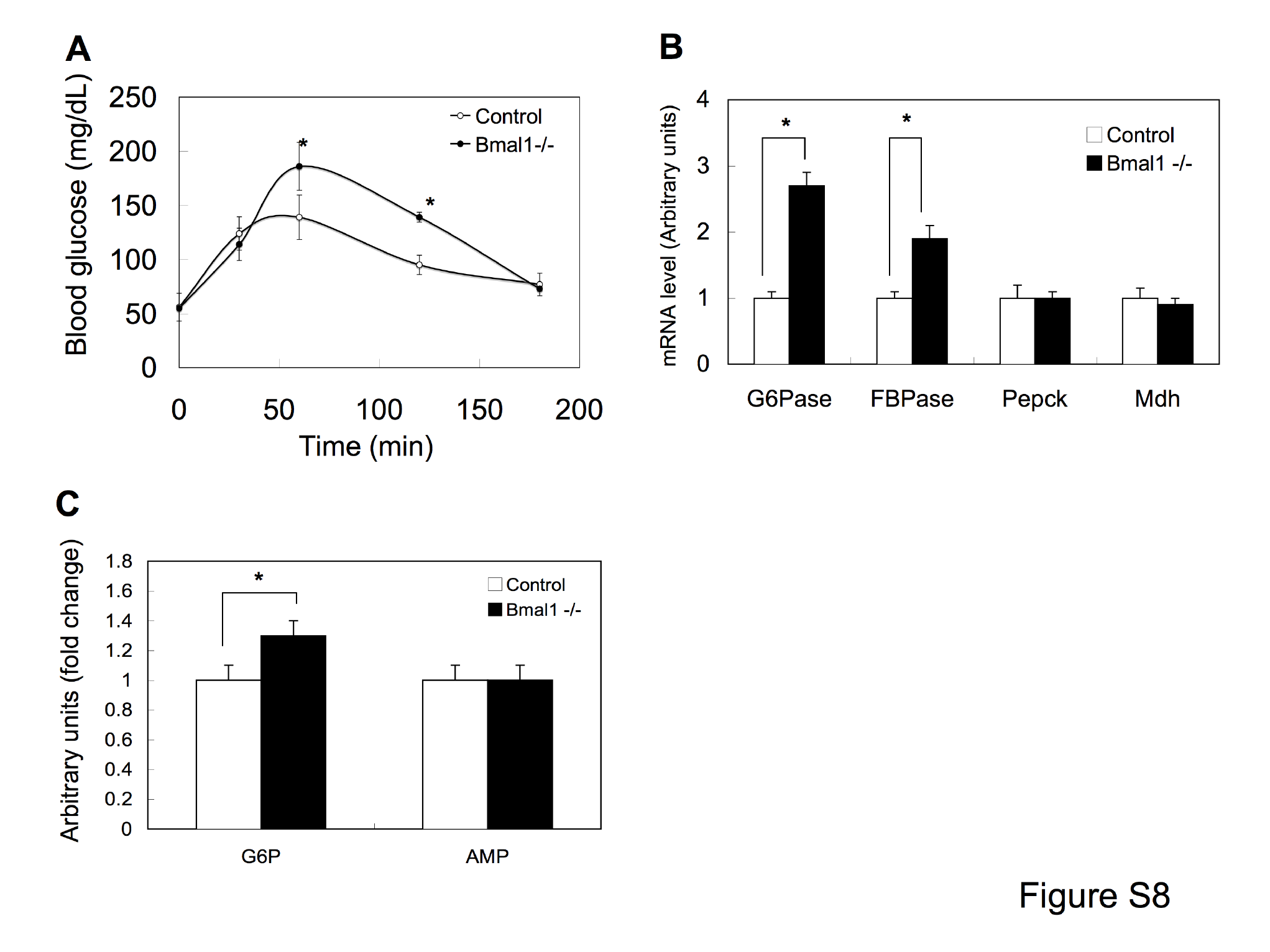

Supplement: Figure S8 — Lowered gluconeogenesis activity in the Bmal1 -/- mice liver. All experiments were performed at ZT10. (A) Male control mice and Bmal1 -/- mice were fasted for 16 h. After i.p. injection of pyruvate (2 g/kg), blood glucose levels were monitored. Data represent the means ± SEM (n = 8 for each genotype and point). Asterisks indicate significant differences (P<0.05). Gene expression levels in the liver of male control and Bmal1 -/- mice were determined by RT-qPCR. Relative mRNA levels were normalized to the 36B4 level. Data represent the means ± SEM (n = 5 for each genotype). Asterisks indicate significant differences (P<0.05). Levels of G6P and AMP in the liver in male control and Bmal1 -/- mice were determined by capillary electrophoresis time-of-flight mass spectrometry. Data represent the means ± SEM (n = 4 for each genotype). Asterisks indicate significant differences (P<0.05). (TIF) [file pone.0025231.s008.tif]

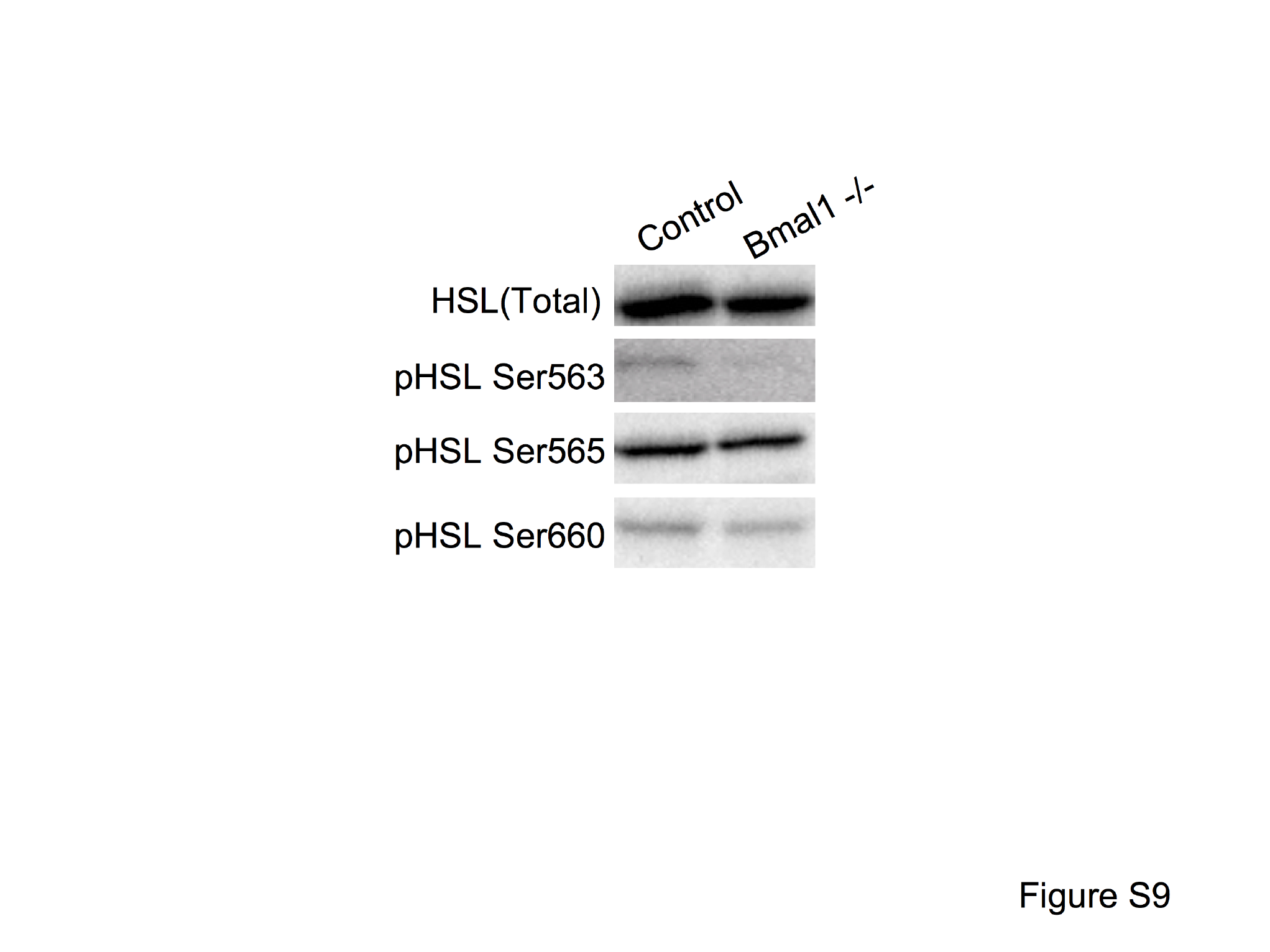

Supplement: Figure S9 — Phosphorylation status of HSL in Bmal1 -/- mice adipose tissue. Total HSL protein level and phosphorylated form of HSL protein in the adipose tissue isolated from male control mice and Bmal1 -/- mice at ZT10 were determined by Western blot. (TIF) [file pone.0025231.s009.tif]
