# Supplementary material for: Deficient of a Clock Gene, Brain and Muscle Arnt-Like Protein-1 (BMAL1), Induces Dyslipidemia and Ectopic Fat Formation
Source: PLoS One. 2011 Sep 22;6(9):e25231. doi: 10.1371/journal.pone.0025231 (PMC3178629; doi:10.1371/journal.pone.0025231)
Supplement: Table S1 — Gene expressions in Bmal1 -/- mice. Gene expressions in control mice and Bmal1 -/- mice were determined by RT-qPCR. Relative mRNA levels were normalized to the 36B4level. N.D. not determined. Each value represented the mean fold-changes (± SEM) of Bmal1 -/- mice in comparison to control mice (n = 5 for each genotype and point). Asterisks indicate significant differences (P<0.05). (DOC) [file pone.0025231.s010.doc]

Table S1. Gene expressions in *Bmal1* -/- mice.

|  | **Liver** | |  | **Muscle** | |
| --- | --- | --- | --- | --- | --- |
|  | **ZT10** | **ZT22** |  | **ZT10** | **ZT22** |
| **Fatty acid binding and transpot** | | | | | |
| Abca1 | 1.5+0.1 | 0.74+0.2 |  | n.d. | n.d. |
| Fatp2 | 1.2+0.3 | 0.9+0.2 |  | n.d. | n.d. |
| Fatp3 | 0.9+0.3 | 0.9+0.3 |  | n.d. | n.d. |
| Fatp4 | 1.0+0.2 | 1.1+0.3 |  | n.d. | n.d. |
| Fatp5 | 0.8+0.2 | 1.1+0.2 |  | n.d. | n.d. |
| Agpat1 | 0.6+0.1 | 0.6+0.2 |  | 0.7+0.2 | 0.9+0.1 |
| Gyk | 0.9+0.1 | 1.1+0.2 |  | 1.6+0.3 | 1.3+0.3 |
| Lipin | 0.8+0.2 | 1.0+0.1 |  | 1.2+0.2 | 1.1+0.2 |
| Ppap2a | 1.0+0.1 | 1.1+0.3 |  | 1.1+0.2 | 1.0+0.2 |
| **Fatty acid oxidation** | | | | | |
| Cpt2 | 1.0+0.2 | 1.2+0.3 |  | 2.4+0.1* | 1.1+0.2 |
| Hadb a | 1.3+0.4 | 1.1+0.2 |  | 1.2+0.2 | 1.2+0.3 |
| Hadb b | 1.1+0.2 | 1.2+0.3 |  | 1.3+0.3 | 1.1+0.3 |
| Adrb2 | n.d. | n.d. |  | 0.4+0.0* | 1.0+0.2 |
| Ucp3 | n.d. | n.d. |  | 1.2+0.3 | 1.0+0.3 |
| **Cholesterol synthesis** | | | | | |
| Cyp51 | 1.0+0.1 | 0.3+0.0* |  | 2.1+0.2* | 1.7+0.2* |
| Dhcr | 1.1+0.1 | 0.5+0.1* |  | 1.6+0.3 | 1.2+0.2 |
| Fdps | 1.1+0.1 | 0.4+0.0* |  | 1.2 + 0.2 | 1.0+0.1 |
| Idi1 | 1.1+0.1 | 0.3+0.1* |  | 1.5+0.1* | 1.3+0.2 |
| Mvk | 1.1+0.1 | 0.4+0.0* |  | 0.9 + 0.0 | 1.2+0.2 |
| Pmvk | 1.0+0.0 | 0.4+0.1* |  | 1.5 + 0.3 | 1.2+0.3 |
| Sc5d | 1.0+0.1 | 0.3+0.0* |  | 1.1 + 0.1 | 1.0+0.2 |
| Sqle | 0.9+0.2 | 0.4+0.0* |  | 1.4 + 0.2 | 0.9+0.2 |

Gene expressions in control mice and *Bmal1 -/-* mice were determined by RT-qPCR. Relative mRNA levels were normalized to the 36B4 level. N.D. not determined. Each value represented the mean fold-changes (+ SEM) of *Bmal1 -/-* mice in comparison to control mice (n=5 for each genotype and point). Asterisks indicate significant differences (*P* < 0.05).
