# Supplementary material for: Deficient of a Clock Gene, Brain and Muscle Arnt-Like Protein-1 (BMAL1), Induces Dyslipidemia and Ectopic Fat Formation
Source: PLoS One. 2011 Sep 22;6(9):e25231. doi: 10.1371/journal.pone.0025231 (PMC3178629; doi:10.1371/journal.pone.0025231)
Supplement: Text S1 — Impaired glucose deposition and acceleration of gluconeogenesis in Bmal1 -/- mice. (DOC) [file pone.0025231.s011.doc]

**Loss of the *Bmal1* gene lowers insulin secretion.**

Lack of proper insulin activity leads to the development of metabolic syndrome. Therefore, to determine the level of insulin activity in *Bmal1* -/- mice, we first conducted oral glucose tolerance tests (OGTT). In both control mice and *Bmal1* -/- mice, the blood glucose levels were elevated and peaked after 30 min of glucose loading (Fig. S7A). However, the disposal rate of blood glucose in *Bmal1* -/- mice was significantly slower than that in the control mice, i.e., after 60 min of administration, the blood glucose level in *Bmal1* -/- mice was almost double that in control mice (Fig. S7A). During OGTT, the serum insulin level in *Bmal1* -/- mice was lower than that in control mice (Fig. S7B). On the other hand, in the presence of an excess amount of insulin, the clearance rate of blood glucose in *Bmal1* -/- mice was similar to that in the control mice (Fig. S7C). Also, *Bmal1* -/- mice had hyperglycemia (Fig. S7D). Interestingly, the insulin content in the pancreas of *Bmal1* -/- mice was approximately 2 times that in the control mice (Fig. S7E). The administration of glibenclamide, a potent stimulator of insulin secretion, increased the plasma insulin level in control mice, but had no effect in *Bmal1* -/- mice (Fig. S7F). Histological analysis revealed that the average size of islets in *Bmal1* -/- mice was almost equal to that in control mice at the age of 12 weeks (Fig. S7G and H). However, at the age of 44 weeks, the islet size in *Bmal1* -/- mice became dramatically smaller than that in control mice (Fig. S7G and H).

**Loss of the *Bmal1* gene stimulates gluconeogenesis in the liver.**

The impaired insulin secretion described in Fig. S7 may alter the activity of glucose metabolism in *Bmal1* -/- mice. Thus, to determine the activity of gluconeogenesis, *Bmal1* -/- mice were subjected to pyruvate challenge experiments. As shown in Fig. S8, the peak blood glucose level was significantly higher and the clearance was slower in *Bmal1* -/- mice, as compared to those in control mice. Among the key enzymes of gluconeogenesis, expression of G6Pase and FBPase in the liver was increased upon *Bmal1* deficiency (Fig. S8B). Also, the level of G6P, a metabolic activator of gluconeogenesis, was significantly higher in the liver of *Bmal1* -/- mice than in that of control mice (Fig. S8C).
